# Depressive Symptoms and Suicidal Ideation in Spanish Medical Students

**DOI:** 10.1192/j.eurpsy.2022.483

**Published:** 2022-09-01

**Authors:** J.A. Blanco, M. Rodriguez, I. Santos Carrasco, M. Queipo De Llano De La Viuda, L. Gallardo Borge, P. Marqués Cabezas, M.J. Mateos Sexmero, J. Blanco Vilches

**Affiliations:** 1 University Clinical Hospital of Valladolid, Psychiatry, Valladolid, Spain; 2 University of Valladolid, Psychiatry, Valladolid, Spain; 3 Clinical Hospital of Valladolid, Psychiatry, Valladolid, Spain

**Keywords:** medical students, Depression, Suicide

## Abstract

**Introduction:**

Medical students have higher rates of depression, anxiety and suicidal ideation over the general population. The onset of these disorders can be a risk factor with unfavorable impact in both medical care and their lives during the years of clinical specialization

**Objectives:**

To assess the prevalence and factors involved in depression, anxiety and suicidal behavior in medical students of the University of Valladolid (Spain). The results are compared with a previous study conducted 5 years earlier

**Methods:**

We used an online self-administered questionnaire that included demographic variables, academic information, sanitary data, Beck Depression Inventory (BDI), Generalized Anxiety Disorder 7 (GAD7), and MINI International Neuropsychiatric Interview for suicide. Chi-Square Test was used for categorical variables, Student`s t-test for quantitative variables and Spearman’s Coefficient to evaluate correlations between variables

**Results:**

362 students of all courses enrolled in Medicine at the University of Valladolid completed the survey. There were no differences between male and female students, both with high rates of moderate-severe depression (27% vs 30,4%), anxiety (42,9% vs 54,5%), and moderate-severe suicide risk (14,2% vs 10,7%). Previous study (n=584) also showed no differences between sexes but with lower rates of moderate-severe depression (14,3% vs 16,3%). 11% reported suicidal thoughts in the past month (11,6% previous study). There was a significant inverse correlation between medical career satisfaction and BDI scores

**Conclusions:**

Five years later, rates of depression and suicide risk could have increased in medical students at the University of Valladolid. We urgently recommend the implementation of mental health prevention programs in this population

**Disclosure:**

No significant relationships.

